# *N*′-Benzoyl-*N*,*N*-diethyl­thio­urea: a monoclinic polymorph

**DOI:** 10.1107/S1600536810009578

**Published:** 2010-03-20

**Authors:** Ligia R. Gomes, Luís M. N. B. F. Santos, João A. P. Coutinho, Bernd Schröder, John Nicolson Low

**Affiliations:** a REQUIMTE, Departamento de Química e Bioquímica, Faculdade de Ciências, Universidade do Porto, Rua do Campo Alegre 687, P-4169_007 Porto, Portugal; b Centro de Investigação em Química, Departamento de Química e Bioquímica, Faculdade de Ciências, Universidade do Porto, Rua do Campo Alegre 687, P-4169_007 Porto, Portugal; c CICECO, Departamento de Química, Universidade de Aveiro, 3810-193 Aveiro, Portugal; d Department of Chemistry, University of Aberdeen, Meston Walk, Old Aberdeen, AB24 3UE, Scotland

## Abstract

In the crystal of the title compound, C_12_H_16_N_2_OS, inversion dimers linked by pairs of N—H⋯S hydrogen bonds occur, generating *R*_2_^2^(8) loops. The mol­ecules are also linked by weak C—H⋯O hydrogen bonds. The structure is isostructural with that of *N*′-benzoyl-*N*,*N*-diethyl­seleno­urea [Bruce *et al.* (2007 ▶). *New J. Chem.* **31**, 1647–1653].

## Related literature

For graph-set notation, see: Bernstein *et al.* (1995 ▶). For the structure of the isomorphous compound *N*,*N*-diethyl-*N*′-benzoyl­seleno­urea, see: Bruce *et al.* (2007 ▶). For a triclinic polymorph of the title compound, see: Bolte & Fink (2003 ▶). For related thio­ureas, see: Braun *et al.* (1987 ▶). For the preparation of the title compound, see: Beyer *et al.* (1975 ▶); Hartmann & Reuther (1973 ▶).
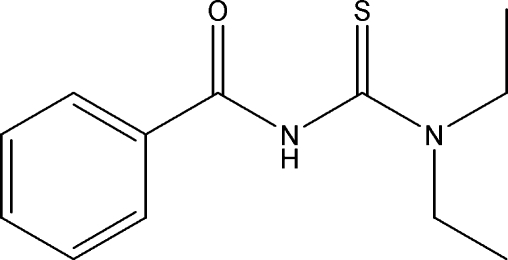


## Experimental

### 

#### Crystal data

C_12_H_16_N_2_OS
*M* *_r_* = 236.33
Monoclinic, 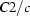

*a* = 20.1727 (7) Å
*b* = 8.4717 (3) Å
*c* = 14.8345 (6) Å
β = 106.553 (2)°
*V* = 2430.11 (16) Å^3^
*Z* = 8
Mo *K*α radiation
μ = 0.25 mm^−1^
*T* = 150 K
0.26 × 0.20 × 0.02 mm

#### Data collection

Bruker SMART APEXII diffractometer
Absorption correction: multi-scan (*SADABS*; Bruker, 2004 ▶) *T*_min_ = 0.939, *T*_max_ = 0.995
18425 measured reflections
3704 independent reflections
2940 reflections with *I* > 2σ(*I*)
*R*_int_ = 0.038

#### Refinement

*R*[*F* ^2^ > 2σ(*F* ^2^)] = 0.036
*wR*(*F* ^2^) = 0.096
*S* = 1.04
3704 reflections
147 parameters
H-atom parameters constrained
Δρ_max_ = 0.41 e Å^−3^
Δρ_min_ = −0.21 e Å^−3^

### 

Data collection: *APEX2* (Bruker, 2004 ▶); cell refinement: *APEX2* and *SAINT* (Bruker, 2004 ▶); data reduction: *SAINT*; program(s) used to solve structure: *SHELXS97* (Sheldrick, 2008 ▶); program(s) used to refine structure: *SHELXL97* (Sheldrick, 2008 ▶); molecular graphics: *ORTEPII* (Johnson, 1976 ▶) and *PLATON* (Spek, 2009 ▶); software used to prepare material for publication: *SHELXL97*.

## Supplementary Material

Crystal structure: contains datablocks global, I. DOI: 10.1107/S1600536810009578/hb5361sup1.cif

Structure factors: contains datablocks I. DOI: 10.1107/S1600536810009578/hb5361Isup2.hkl

Additional supplementary materials:  crystallographic information; 3D view; checkCIF report

## Figures and Tables

**Fig. 1.**
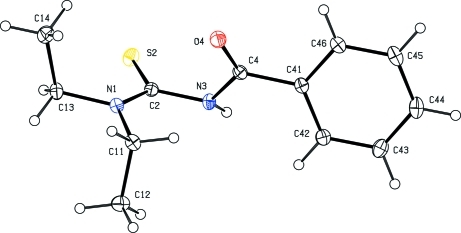
A view of (I) with displacement ellipsoids drawn at the 30% probability level.

**Fig. 2.**
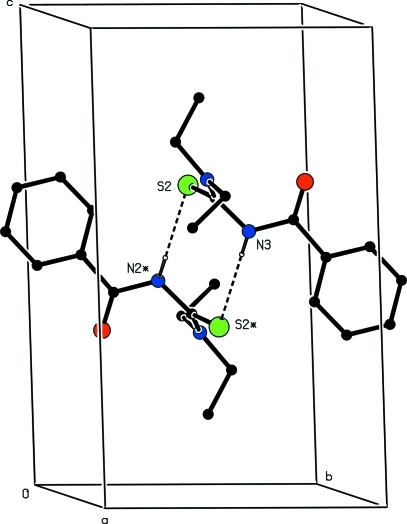
A view of the R22(8) dimer lying across the centre-of symmetry at (0.5,0.5,0.5). Atoms marked with an asterisk,*, are in the molecule at (1-x,1-y,1-z). Hydrogen atoms not involved in the hydrogen bonding are omitted for the sake of clarity.

## supplementary crystallographic information

### Comment

The molecule of the title compound, (I) is shown in Fig.1, the bond lengths and angles show no unusual features. The structure is isostructural with that of *N*,*N*-diethyl-*N*'-benzoylselenourea, Bruce *et al.*, 2007. An N3—H3···S2(1-x,1-y,1-z) hydrogen bond links the molecules into R^2^_2_(8), Bernstein *et al.*, 1995, centrosymetric dimers across the crystallographic centre of symmetry at (0.5, 0.5, 0.5). The bond lengths involved are N3—H3, 0.91 Å, H3···S2, 2.56Å and N3···S2, 3.4595 (11)Å and the angle at H3 is 169°, Fig. 2. The dimers are linked together to form sheets which lie parallel to (-101) by the weak C13—H13..O4(1.5-x,-0.5+y,1.5-z) hydrogen bond with C13—H13B, 0.99 Å, H13B···.O2, 2.59 Å, C13···.O4, 3.3594 (18)Å and an angle at H13B of 135°. This sheet is further re-inforced by a πi···πi interaction involving the phenyl rings at (x, y, z) and (1-x, 2-y, 1-z) which have a centre-to-centre distance of 4.3861 (8) Å, a perpendicular spacing of 3.5511 (6)Å and a slippage of 2.574 Å.

The structure of another polymorph of (1) is deposited as a private communication in the CCDC database, Bolte & Fink (2003). This is reported as crystallising in spacegroup P1 with four molecules in the asymmetric unit. In this compound the molecules are linked into two sets of C4 chains by N—H···O hydrogen bonds. These chains are formed by hydrogen bonded pairs of molecules in which the the N1—C2—N3—C4 torsion angles (our) numbering) are 78.6 (4)° and -80.8 (3)° in one pair and 78.7 (4)° and -81.9 (3)°. In these conformations the O atom is in a favourable position for forming N—H···O hydrogen bonds. In (1) the N1—C2—N3—C4 torsion angle is -71.43 (14)° and the S atom then becomes more accessible as an acceptor for a hydrogen bond and the O atom less so. Related thiourea structures are discussed in Braun *et al.*, (1987).

### Experimental

The title compound was prepared as described by Hartmann & Reuther (1973) and Beyer *et al.* (1975). The reaction as described in these papers produced yellow plates of (I), which after washing in ethanol at room temperature, were suitable for X-ray diffraction without recrystallisation.

### Refinement

H atoms were treated as riding atoms with C—H(aromatic), 0.95 Å, C—H(CH_2_), 0.99 Å. The atom attached to N1 was located on a difference map at a distance of 0.9138Å and was fixed as a riding atom at this distance.

### Crystal data

**Table:**

C_12_H_16_N_2_OS	*F*(000) = 1008
*M**_r_* = 236.33	*D*_x_ = 1.292 Mg m^−^^3^
Monoclinic, *C*2/*c*	Mo *K*α radiation, λ = 0.71073 Å
*a* = 20.1727 (7) Å	Cell parameters from 173 reflections
*b* = 8.4717 (3) Å	θ = 2.0–28.2°
*c* = 14.8345 (6) Å	µ = 0.25 mm^−^^1^
β = 106.553 (2)°	*T* = 150 K
*V* = 2430.11 (16) Å^3^	Plate, yellow
*Z* = 8	0.26 × 0.20 × 0.02 mm

### Data collection

**Table:**

Bruker SMART APEXII diffractometer	3704 independent reflections
Radiation source: fine-focus sealed tube	2940 reflections with *I* > 2σ(*I*)
graphite	*R*_int_ = 0.038
ω scans	θ_max_ = 30.5°, θ_min_ = 3.9°
Absorption correction: multi-scan (*SADABS*; Bruker, 2004)	*h* = −28→28
*T*_min_ = 0.939, *T*_max_ = 0.995	*k* = −12→12
18425 measured reflections	*l* = −20→21

### Refinement

**Table:**

Refinement on *F*^2^	Primary atom site location: structure-invariant direct methods
Least-squares matrix: full	Secondary atom site location: difference Fourier map
*R*[*F*^2^ > 2σ(*F*^2^)] = 0.036	Hydrogen site location: inferred from neighbouring sites
*wR*(*F*^2^) = 0.096	H-atom parameters constrained
*S* = 1.04	*w* = 1/[σ^2^(*F*_o_^2^) + (0.041*P*)^2^ + 1.4589*P*] where *P* = (*F*_o_^2^ + 2*F*_c_^2^)/3
3704 reflections	(Δ/σ)_max_ < 0.001
147 parameters	Δρ_max_ = 0.41 e Å^−^^3^
0 restraints	Δρ_min_ = −0.21 e Å^−^^3^

### Special details

**Table:**

Geometry. All esds (except the esd in the dihedral angle between two l.s. planes) are estimated using the full covariance matrix. The cell esds are taken into account individually in the estimation of esds in distances, angles and torsion angles; correlations between esds in cell parameters are only used when they are defined by crystal symmetry. An approximate (isotropic) treatment of cell esds is used for estimating esds involving l.s. planes.
Refinement. Refinement of *F*^2^ against ALL reflections. The weighted *R*-factor wR and goodness of fit *S* are based on *F*^2^, conventional *R*-factors *R* are based on *F*, with *F* set to zero for negative *F*^2^. The threshold expression of *F*^2^ > σ(*F*^2^) is used only for calculating *R*-factors(gt) etc. and is not relevant to the choice of reflections for refinement. *R*-factors based on *F*^2^ are statistically about twice as large as those based on *F*, and *R*- factors based on ALL data will be even larger.

### Fractional atomic coordinates and isotropic or equivalent isotropic displacement parameters (Å^2^)

**Table:**

	*x*	*y*	*z*	*U*_iso_*/*U*_eq_	
S2	0.533573 (16)	0.44430 (4)	0.64948 (2)	0.02564 (9)	
O4	0.64408 (5)	0.83285 (11)	0.66081 (6)	0.0268 (2)	
N1	0.66786 (5)	0.47946 (12)	0.66756 (7)	0.0196 (2)	
N3	0.59450 (5)	0.64040 (11)	0.55563 (7)	0.0195 (2)	
H3	0.5563	0.6221	0.5060	0.023*	
C2	0.60315 (6)	0.52200 (13)	0.62564 (8)	0.0187 (2)	
C4	0.61335 (6)	0.79534 (14)	0.58044 (9)	0.0197 (2)	
C11	0.72802 (6)	0.52699 (14)	0.63579 (9)	0.0219 (2)	
H11A	0.7689	0.5404	0.6910	0.026*	
H11B	0.7183	0.6295	0.6026	0.026*	
C12	0.74337 (7)	0.40383 (16)	0.57050 (10)	0.0281 (3)	
H12A	0.7556	0.3039	0.6045	0.042*	
H12B	0.7821	0.4395	0.5480	0.042*	
H12C	0.7024	0.3884	0.5168	0.042*	
C13	0.68400 (7)	0.36738 (14)	0.74658 (9)	0.0233 (2)	
H13A	0.7264	0.3079	0.7474	0.028*	
H13B	0.6456	0.2908	0.7380	0.028*	
C14	0.69465 (8)	0.45271 (17)	0.83921 (10)	0.0320 (3)	
H14A	0.7331	0.5274	0.8481	0.048*	
H14B	0.7054	0.3759	0.8907	0.048*	
H14C	0.6524	0.5101	0.8388	0.048*	
C41	0.59558 (6)	0.91116 (14)	0.50117 (9)	0.0193 (2)	
C42	0.58864 (6)	0.86700 (15)	0.40819 (9)	0.0226 (2)	
H42	0.5927	0.7592	0.3930	0.027*	
C43	0.57585 (7)	0.98072 (17)	0.33807 (10)	0.0281 (3)	
H43	0.5724	0.9510	0.2751	0.034*	
C44	0.56814 (7)	1.13787 (16)	0.35958 (10)	0.0297 (3)	
H44	0.5589	1.2153	0.3113	0.036*	
C45	0.57385 (6)	1.18181 (15)	0.45143 (10)	0.0275 (3)	
H45	0.5678	1.2892	0.4658	0.033*	
C46	0.58840 (6)	1.06976 (14)	0.52255 (9)	0.0234 (2)	
H46	0.5935	1.1008	0.5857	0.028*	

### Atomic displacement parameters (Å^2^)

**Table:**

	*U*^11^	*U*^22^	*U*^33^	*U*^12^	*U*^13^	*U*^23^
S2	0.02011 (15)	0.03108 (17)	0.02609 (17)	−0.00359 (12)	0.00715 (12)	0.00515 (13)
O4	0.0307 (5)	0.0238 (4)	0.0221 (4)	−0.0002 (4)	0.0016 (4)	−0.0030 (4)
N1	0.0191 (5)	0.0180 (5)	0.0211 (5)	0.0003 (4)	0.0047 (4)	0.0009 (4)
N3	0.0189 (5)	0.0180 (5)	0.0192 (5)	−0.0019 (4)	0.0016 (4)	0.0018 (4)
C2	0.0202 (5)	0.0171 (5)	0.0179 (5)	−0.0012 (4)	0.0043 (4)	−0.0013 (4)
C4	0.0169 (5)	0.0191 (5)	0.0232 (6)	0.0005 (4)	0.0057 (4)	−0.0003 (4)
C11	0.0170 (5)	0.0203 (6)	0.0276 (6)	−0.0017 (4)	0.0051 (5)	−0.0005 (5)
C12	0.0293 (6)	0.0278 (6)	0.0292 (7)	−0.0031 (5)	0.0117 (5)	−0.0042 (5)
C13	0.0250 (6)	0.0195 (5)	0.0228 (6)	0.0013 (4)	0.0028 (5)	0.0037 (5)
C14	0.0407 (8)	0.0303 (7)	0.0235 (6)	0.0024 (6)	0.0070 (6)	0.0005 (5)
C41	0.0146 (5)	0.0191 (5)	0.0234 (6)	−0.0005 (4)	0.0041 (4)	0.0010 (4)
C42	0.0215 (5)	0.0210 (6)	0.0247 (6)	−0.0016 (4)	0.0056 (5)	0.0004 (5)
C43	0.0264 (6)	0.0322 (7)	0.0231 (6)	−0.0027 (5)	0.0027 (5)	0.0032 (5)
C44	0.0242 (6)	0.0278 (7)	0.0328 (7)	−0.0012 (5)	0.0011 (5)	0.0097 (5)
C45	0.0210 (6)	0.0195 (6)	0.0393 (7)	0.0012 (5)	0.0040 (5)	0.0030 (5)
C46	0.0205 (5)	0.0202 (6)	0.0282 (6)	0.0005 (4)	0.0050 (5)	−0.0011 (5)

### Geometric parameters (Å, °)

**Table:**

S2—C2	1.6767 (12)	C13—H13A	0.9900
O4—C4	1.2188 (15)	C13—H13B	0.9900
N1—C2	1.3258 (15)	C14—H14A	0.9800
N1—C13	1.4712 (15)	C14—H14B	0.9800
N1—C11	1.4774 (15)	C14—H14C	0.9800
N3—C4	1.3869 (15)	C41—C42	1.3971 (17)
N3—C2	1.4183 (15)	C41—C46	1.3975 (16)
N3—H3	0.9138	C42—C43	1.3871 (18)
C4—C41	1.4946 (17)	C42—H42	0.9500
C11—C12	1.5145 (17)	C43—C44	1.388 (2)
C11—H11A	0.9900	C43—H43	0.9500
C11—H11B	0.9900	C44—C45	1.385 (2)
C12—H12A	0.9800	C44—H44	0.9500
C12—H12B	0.9800	C45—C46	1.3870 (18)
C12—H12C	0.9800	C45—H45	0.9500
C13—C14	1.5129 (18)	C46—H46	0.9500
			
C2—N1—C13	120.93 (10)	N1—C13—H13B	109.5
C2—N1—C11	124.38 (10)	C14—C13—H13B	109.5
C13—N1—C11	114.50 (10)	H13A—C13—H13B	108.0
C4—N3—C2	120.52 (10)	C13—C14—H14A	109.5
C4—N3—H3	118.6	C13—C14—H14B	109.5
C2—N3—H3	111.7	H14A—C14—H14B	109.5
N1—C2—N3	115.79 (10)	C13—C14—H14C	109.5
N1—C2—S2	124.46 (9)	H14A—C14—H14C	109.5
N3—C2—S2	119.75 (8)	H14B—C14—H14C	109.5
O4—C4—N3	122.07 (11)	C42—C41—C46	119.59 (11)
O4—C4—C41	122.66 (11)	C42—C41—C4	122.35 (11)
N3—C4—C41	115.24 (10)	C46—C41—C4	118.02 (11)
N1—C11—C12	110.64 (10)	C43—C42—C41	119.96 (12)
N1—C11—H11A	109.5	C43—C42—H42	120.0
C12—C11—H11A	109.5	C41—C42—H42	120.0
N1—C11—H11B	109.5	C42—C43—C44	120.19 (13)
C12—C11—H11B	109.5	C42—C43—H43	119.9
H11A—C11—H11B	108.1	C44—C43—H43	119.9
C11—C12—H12A	109.5	C45—C44—C43	120.03 (13)
C11—C12—H12B	109.5	C45—C44—H44	120.0
H12A—C12—H12B	109.5	C43—C44—H44	120.0
C11—C12—H12C	109.5	C44—C45—C46	120.30 (12)
H12A—C12—H12C	109.5	C44—C45—H45	119.9
H12B—C12—H12C	109.5	C46—C45—H45	119.9
N1—C13—C14	110.93 (10)	C45—C46—C41	119.90 (12)
N1—C13—H13A	109.5	C45—C46—H46	120.0
C14—C13—H13A	109.5	C41—C46—H46	120.0
			
C13—N1—C2—N3	174.96 (10)	O4—C4—C41—C42	151.78 (12)
C11—N1—C2—N3	−10.38 (16)	N3—C4—C41—C42	−26.09 (16)
C13—N1—C2—S2	−5.74 (16)	O4—C4—C41—C46	−25.75 (17)
C11—N1—C2—S2	168.92 (9)	N3—C4—C41—C46	156.38 (10)
C4—N3—C2—N1	−71.43 (14)	C46—C41—C42—C43	1.06 (17)
C4—N3—C2—S2	109.23 (11)	C4—C41—C42—C43	−176.44 (11)
C2—N3—C4—O4	7.58 (17)	C41—C42—C43—C44	−1.66 (19)
C2—N3—C4—C41	−174.54 (10)	C42—C43—C44—C45	0.60 (19)
C2—N1—C11—C12	−93.09 (14)	C43—C44—C45—C46	1.07 (19)
C13—N1—C11—C12	81.88 (13)	C44—C45—C46—C41	−1.66 (18)
C2—N1—C13—C14	−88.47 (14)	C42—C41—C46—C45	0.59 (17)
C11—N1—C13—C14	96.37 (13)	C4—C41—C46—C45	178.19 (11)

### Hydrogen-bond geometry (Å, °)

**Table:**

*D*—H···*A*	*D*—H	H···*A*	*D*···*A*	*D*—H···*A*
N3—H3···S2^i^	0.91	2.56	3.4595 (11)	169
C13—H13A···O4^ii^	0.99	2.59	3.3594 (18)	135

Symmetry codes: (i) −*x*+1, −*y*+1, −*z*+1; (ii) −*x*+3/2, *y*−1/2, −*z*+3/2.
